# Adenosine A_2A_ receptor stimulation restores cell functions and differentiation in Niemann-Pick type C-like oligodendrocytes

**DOI:** 10.1038/s41598-019-46268-8

**Published:** 2019-07-05

**Authors:** Chiara De Nuccio, Antonietta Bernardo, Antonella Ferrante, Rita Pepponi, Alberto Martire, Mario Falchi, Sergio Visentin, Patrizia Popoli, Luisa Minghetti

**Affiliations:** 1 Research Coordination and Support Service, Istituto Superiore di Sanità, Viale Regina Elena 299, 00161 Rome, Italy; 2 National Center for Research and Preclinical and Clinical Evaluation of Drugs, Istituto Superiore di Sanità, Viale Regina Elena 299, 00161 Rome, Italy; 3 National Research Center on HIV/AIDS, Istituto Superiore di Sanità, Viale Regina Elena 299, 00161 Rome, Italy

**Keywords:** Neuroscience, Diseases of the nervous system

## Abstract

Niemann Pick type C (NPC) disease is a rare neurovisceral disorder. Mutations in *npc1* gene induce an intracellular accumulation of unesterified cholesterol in the endosomal/lysosomal system causing cell death. We recently showed that stimulation of adenosine A_2A_ receptors (A_2A_R) restores cholesterol accumulation in late endosomes/lysosomes in human NPC fibroblasts and neural cell lines transiently transfected with NPC1 siRNA, suggesting that these receptors might be targeted to contrast the disease. Since NPC1 disease is characterized by dysmyelination and maturational arrest of oligodendrocyte progenitors (OPs), in this study, we investigated whether A_2A_R stimulation could promote oligodendrocyte differentiation and myelin formation, thus overcoming these important neurological abnormalities. We developed a NPC1 pharmacological model, in which primary cultures of OPs are exposed to a cholesterol transport inhibitor to induce a NPC1-like phenotype characterized by several typical features such as (i) cholesterol accumulation, (ii) altered mitochondrial morphology and membrane potential, (iii) defect of autophagy and (iv) maturation arrest. The A_2A_R agonist CGS21680 normalized all NPC1-like features. The ability of CGS21680 of rescuing OP from maturational arrest and promoting their differentiation to mature OL, suggests that A_2A_R stimulation might be exploited to correct dysmyelination in NPC1, further supporting their therapeutic potential in the disease.

## Introduction

Niemann-Pick type C 1 disease (NPC1) is an autosomal recessive and progressive neurovisceral disorder. NPC1 is caused by mutations in the *npc1* gene, which primarily lead to abnormal cholesterol trafficking and to intracellular accumulation of unesterified cholesterol and glycosphingolipids in late endosomes and lysosomes, as well as to other cellular abnormalities, such as mitochondrial impairment and disruption of calcium and autophagy homeostasis^1–3^. The most severe consequences of NPC1-mutation occur in the CNS and include neurodegeneration, neuroinflammation and dysmyelination^4–6^.

The myelin defects observed in both patients and NPC mouse models likely arise either from a defective differentiation of oligodendrocytes (OL), the myelin forming cells of the CNS, or a failure of proper axon-glial interaction^6–9^. Mitochondria are crucial organelles for OL differentiation and myelin formation^10–12^ and defective mitochondrial functions have been observed in NPC animal and cell models^3,13–16^.

We have previously shown that the stimulation of adenosine A_2A_ receptors (A_2A_R) is able to rescue the abnormal phenotype of genetic NPC models. In particular, we demonstrated that A_2A_R stimulation restores calcium homeostasis, mitochondrial membrane potential (mMP) and cholesterol accumulation in fibroblasts from NPC1 patients and in human neuronal and oligodendroglial NPC1 cell lines (i.e. neuroblastoma SH-SY5Y and oligodendroglial MO3.13) transiently transfected with NPC1 small interfering RNA^15,16^.

These models were very useful to provide a clear *in vitro* proof of concept that A_2A_R agonists are promising potential drugs for NPC1 disease. However, the siNPC1-MO3.13 model did not allow ascertaining whether the rescued NPC1 oligodendroglia could complete their maturation to myelin producing cells, since the down-regulation of the NPC1 was transient and maturation of MO3.13 needs exposure to differentiating agents for several days^16^.

To gain insight on the ability of A_2A_R agonists to overcome the maturation arrest in NPC1 oligodendrocyte, which prevents a correct myelination, we switched to primary cultures of OL progenitors (OPs) exposed to U18666a, a cholesterol transport inhibitor known to induce a NPC-like phenotype in a variety of cell types^17^. Differentiation of OPs occurs spontaneously and closely recapitulates the *in vivo* situation, in a period compatible with our experimental conditions. Here we show that U18666a-treated OPs exhibited cholesterol accumulation, altered mMP and mitochondrial morphology, autophagy flux block and maturation arrest. In line with our previous results, A_2A_R-activation by a specific agonist (CGS21680) rescues the NPC1 phenotype, but for the first time, we demonstrate that A_2A_R-activation overcomes the OP maturation arrest. These results support the hypothesis that A_2A_R stimulation could counteract dysmyelination providing a solid basis for future *in vivo* experiments, and making such a receptor potentially suitable therapeutic target for NPC1 disease.

## Results

### The A_2A_ agonist CGS21680 rescues U18666a-induced cholesterol accumulation and mitochondrial abnormalities in rat primary OP cultures

To establish a pharmacological NPC1 model, highly purified OP cultures, after 1 day *in vitro*, were treated for 48 h with U18666a.

**Figure 1:**
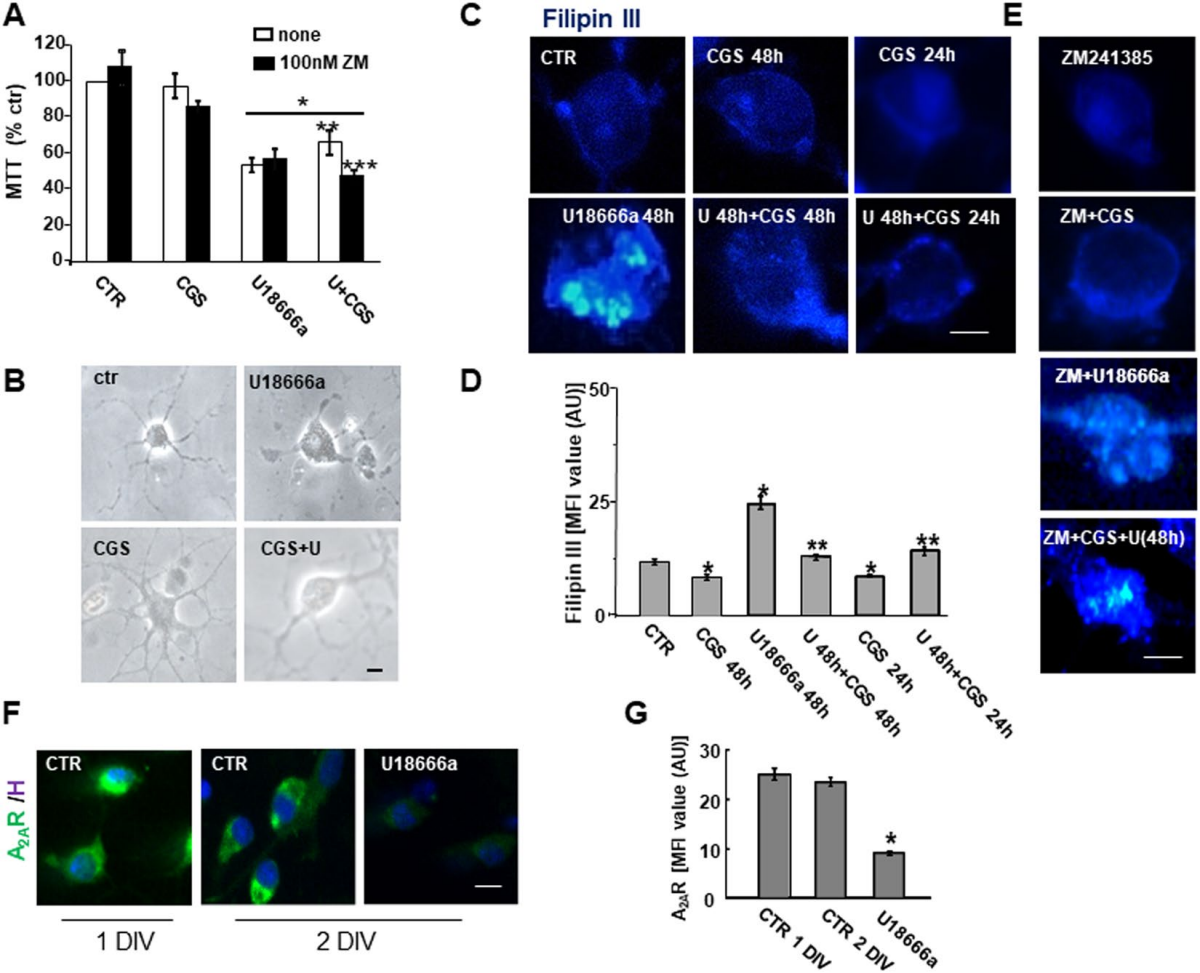
The A_2A_R agonist CGS21680 rescues the effects of U18666a on viability, morphology and cholesterol accumulation. (**A**) Cell viability was assessed by MTT reduction assay in OPs, at 3 DIV, treated for 48 h with 1.25 μM of U18666a (U), 100 nM of CGS21680 (CGS) and 100 nM of ZM241385 (ZM). Data are presented as mean percentages of control cultures ± SEM, n = 4–6 independent experiments run in triplicate (*p < 0.001 vs CTR **p < 0.01 vs U ***p < 0.05 vs U + CGS). (**B**) Phase contrast morphology of cultured OPs treated for 48 h with U18666a and CGS21680 were shown. Scale bar = 50 µm (**C**–**E**). Cholesterol was labeled with Filipin III (blue). (**C**) The OPs were treated for 48 h with U18666a alone or with CGS21680. CGS21680 was added together with U18666a or in the last 24 h of treatment. Mean fluorescence intensities (MFI value) of Filipin III are shown in D. Data are mean ± SEM, n = 150 cells for condition (*p < 0.001 vs CTR **p < 0.001 vs U). (**E**) Filipin III was evaluated in cultures treated with A_2A_R antagonist ZM241385 (100 nM), added 30 min earlier than the other drugs. (**F**) A_2A_R expression in OP, at 1 and 2 DIV, treated or not for 24 h with U18666a at 1DIV. (**G**) Mean fluorescence intensities (MFI value) were shown. Data are mean ± SEM of n. 150 cells for condition (*p < 0.005 vs CTR. Scale bar = 50 µm).

Cell viability of OPs exposed to U18666a (1.25 μg/mL) for 48 h was reduced by about 50% as determined by the MTT assay (Fig. 1A). The presence of the A_2A_R agonist CGS21680 protected OPs against this toxic effect and its activity was prevented by the A_2A_R antagonist ZM241385 (100 nM), supporting a specific involvement of A_2A_Rs (Fig. 1A). U18666a treatment induced an evident cholesterol accumulation and an abnormal morphology characterized by the presence of vacuole-like structures (Fig. 1B,C). Cholesterol accumulation was determined by using Filipin III. U18666a induced cholesterol accumulation in vacuole-like formations and a significant increase in the mean fluorescence intensity (MFI) of Filipin III as compared to untreated cells (Fig. 1C,D). Cellular vacuolization and cholesterol accumulation were significantly reduced when OPs were co-treated with U18666a and the A_2A_R agonist CGS21680 (100 nM, Fig. 1B–D). The reduction of cholesterol accumulation by CGS21680 occurred also when CGS21680 was added during the last 24 h of U18666a treatment, suggesting that the protective mechanisms triggered by CGS21680 are effective even when the damaging effects of U18666a are already in place (Fig. 1C,D). The presence of the A_2A_R antagonist ZM241385 blocked the effect of CGS21680 on U18666a-induced cholesterol accumulation, while alone it did not affect control, CGS21680- or U18666a-treated cells (Fig. 1E). We also tested BAY 60-6583, a selective A_2B_R agonist, on cholesterol accumulation induced by U18666a. Unlike CGS21680, BAY 60-6583 (10 μM^18^) did not reduce the cholesterol accumulation, ruling out the involvement of A_2B_R and in line with selective effects of A_2A_R activation on rescuing NPC1 phenotype (Fig. 1S).

**Figure 2:**
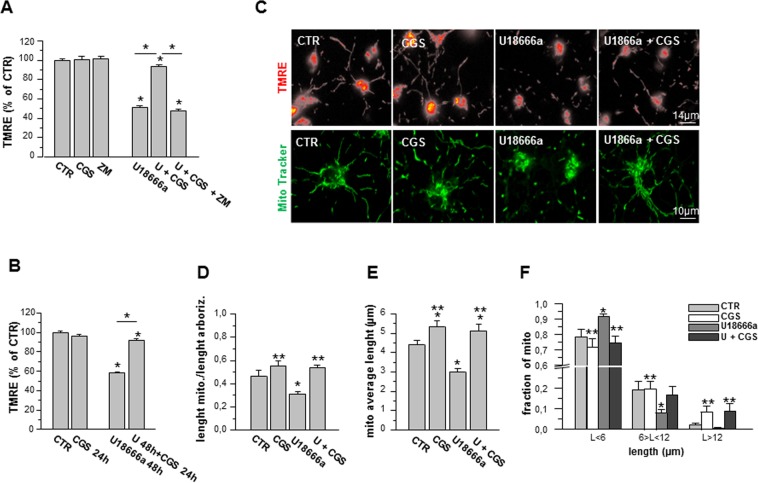
The effect of U18666a on mitochondria is rescued by A_2A_R stimulation. (**A**,**B**) The mitochondrial membrane potential (mMP) was measured by fluorescent potentiometric dye TMRE. Average of the percentage of TMRE-fluorescence intensity was calculated in mitochondria of OPs treated for 48 h with U18666a (U), CGS21680 (CGS) and ZM241385 (ZM) and in cell treated for 48 h with U alone or in combination with CGS, added during the last 24 h of incubation. Data are mean  ± SEM of 6 independent cell preparations, from n = 80 to n = 240 mitochondria for each single condition; CTR is taken as 100% (*p < 0.05). (**C**) Examples of OPs loaded with TMRE (top) or with MitoTracker (bottom) under different culture conditions (Scale bar = 14 and 10 µm). (**D**) Evaluation of mitochondrial density per unit of length: MitoTracker emission was measured in cell processes by line profile analysis. The total length of mitochondria was measured on the total length of all line profiles drawn on arborization in bright-field images. (**E**) Mitochondrial average length was measured in 50 cell processes (from 178 to 215 mitochondria) per condition. (**F**) For each condition, the mitochondria were divided into three categories, based on their size (<6 μm, between 6 and 12 μm, and >12 μm). Data are mean ± SEM of 3 independent cell preparations (*p < 0.05 vs CTR. **p < 0.05 vs U).

Since A_2A_R expression in OLs is known to be developmentally regulated, and being mainly expressed at the immature stages of development^19^ (Fig. 2S), we evaluated by immunofluorescence the effects of U18666a on A_2A_R expression. As Fig. 1F,G show, A_2A_R were highly expressed in OPs at 1 and 2 days *in vitro* (1-2 DIV) and the level of expression was significantly decreased after 24 h treatment with U18666a (to less than 65% of untreated cells). Nonetheless, this level of expression was sufficient to trigger the protective mechanism against cholesterol accumulation.

In order to verify if mitochondrial membrane potential (mMP) was affected as in NPC1, we performed TMRE analyses. As shown in Fig. 2A, OPs treated with U18666a (1.25 μg/mL, 48 h), showed a reduction of about 50% in TMRE fluorescence intensity with respect to CTR cells, indicating a lower mMP, which has been described as a typical characteristic of NPC1-like phenotype^20^. mMP amplitude was increased by CGS21680 and ZM241385 abolished the effect of the agonist, supporting the specific involvement of A_2A_R. As described for cholesterol accumulation, CGS21680 added during the last 24 h reverted the depolarization of mMP induced by 48 h treatment with U18666a (Fig. 2B).

To determine whether U18666a affects mitochondrial network morphology, OP cultures were stained with MitoTracker probe (Fig. 2C). To quantify mitochondrial density per unit of length, we measured MitoTracker fluorescence changes along the entire arborization of single OPs. The treatment with U18666a induced a reduction of mitochondrial density per unit of length and mitochondrial average length. CGS21680 abolished the effect of U18666a on both mitochondrial density per unit of length and mitochondrial average length (Fig. 2D,E). In Fig. 2F, for each condition, mitochondria were divided into three categories, based on their length (<6 μm, >6 and <12 μm, and >12 μm). U18666a increased the fraction of short mitochondria and decreased those of longer mitochondria, in line with the disrupted mitochondrial homeostasis typical of NPC1^13^. CGS21680 restored the ratio among the three length categories to control conditions.

### The A_2A_R agonist CGS21680 regulates the autophagic flux in U18666a-treated primary OP cultures

A mounting body of evidence suggests a defect of autophagy in NPC1 models^21^. To assess whether U18666a induced an unbalance in autophagic flux, we considered three proteins involved in different stages of autophagy as Beclin 1, microtubule-associated protein 1 light chain 3 (LC3) and lysosome-associated membrane glycoprotein 2 (LAMP2).

**Figure 3:**
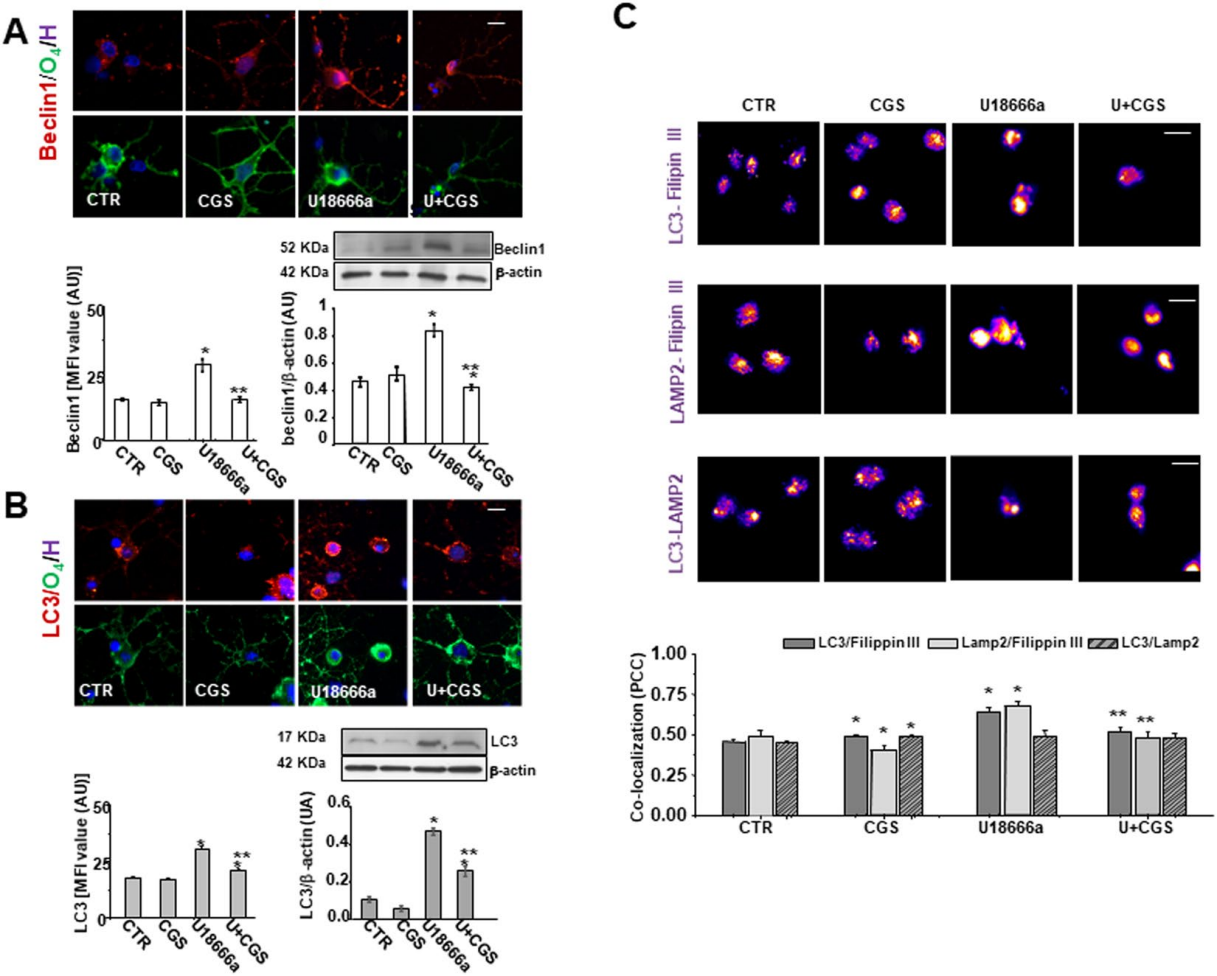
CGS21680 modulates Beclin 1 and LC3 expression, which are upregulated by U18666a and the formation of cholesterol-loaded autophagosome and lysosome. (**A**,**B**) OPs treated for 48 h with U18666a (U) and CGS21680 (CGS) were immunostained for Beclin 1 and LC3 as markers of early stages of autophagy. Examples of OPs immunostained with Beclin 1 or LC3 (red) and with O_4_ (green) are given. Nuclei were stained using the Hoechst 33258 nuclear fluorochrome (blue). Scale bar = 50 µm. Mean fluorescence intensities (MFI value) were shown (data are mean ± SEM of n = 100–150 cells for condition). Western blot analysis of OP cultures treated as before were shown. β-actin expression was used to normalize Beclin 1 and LC3 expression (bar graphs). Representative western blots of three different experiments were shown. Full-length blots are presented in Supplementary Fig. 3S. Data are mean ± SEM (*p < 0.005 vs CTR; **p < 0.05 vs U). (**C**) After treatment with U and CGS for 48 h, the cells were fixed and labeled with anti-LC3 and Filipin III, anti-LAMP2 and Filipin III and with LAMP2 and LC3. The co-localization between these markers was evaluated. Images represent co-localization maps of red/blue channels using ImageJ software (see Material and Methods section). The degree of co-localization was estimated by Pearson’s coefficient (PCC). Data are mean ± SEM of n. 120–150 cells for each condition, of which it was evaluated co-localization (*p < 0.005 vs CTR; **p < 0.05 vs U. Scale bar = 50 µm).

Beclin 1 is a cytoplasmic protein necessary for the formation of autophagosomes, found in the early stages of autophagy. Immunofluorescence staining of Beclin 1 revealed a dotted pattern, with a uniform cell distribution (cellular morphology is shown by O_4_ immunofluorescence). As shown in Fig. 3A, U18666a induced a significant increase in Beclin 1 levels with respect to control and CGS21680 significantly reduced the effect. These results were confirmed by western blot analysis. As for Beclin 1, U18666a treatment increased LC3 levels and such effect was reduced to control levels by CGS21680, as shown by immunofluorescence and western blot analysis (Fig. 3B). Again, LAMP2 expression levels, which is used to detect late endosomal and lysosomal compartments, was increased in U18666a-treated OPs (MFI U18666a: 47.2 ± 3.37; MFI CTR: 20.4 ± 1.3; p < 0.0001). The A_2A_R agonist alone had no significant effect (MFI CGS21680: 17.4 ± 0.58), but reduced the effect of U18666a (MFI U + CGS21680: 34.9 ± 1.9; p < 0.005).

Cholesterol accumulation in autophagosomal and lysosomal compartments was evaluated by analyzing the co-localization of Filipin III with LC3 or LAMP2 labeling (see Material and Methods section) (Fig. 3C). The analyses showed the highest co-localization of cholesterol and each of the two markers in U18666a-treated OPs. The presence of CGS21680 attenuated such co-localizations. The A_2A_R agonist by itself slightly but significantly increased co-localization of LC3 and Filipin III and decreased that of LAMP2 and Filipin III.

The highest co-localization of LAMP2 and Filipin III confirms the cholesterol accumulation in lysosome compartment, a pathological marker of NPC disease. Since the clearance of autophagosomes occurs via fusion with lysosomes, we evaluated the co-localization of LC3 and LAMP2. Interestingly, in spite of the increased levels of autophagosomes (LC3) and of lysosomes (LAMP2), LC3/LAMP2 co-localization in U18666a-treated OPs was not increased, suggesting that the progression of autophagic flux was impaired. These results suggest that the activation of A_2A_R protects U18666a-treated OPs by preventing the abnormal formation of cholesterol-loaded autophagosomes (LC3 positive) and lysosomes (LAMP2 positive) and the unbalance in autophagic flux in U18666a-treated OPs.

### The A_2A_R activation overcomes the maturation arrest in U18666a-treated primary OP cultures

**Figure 4:**
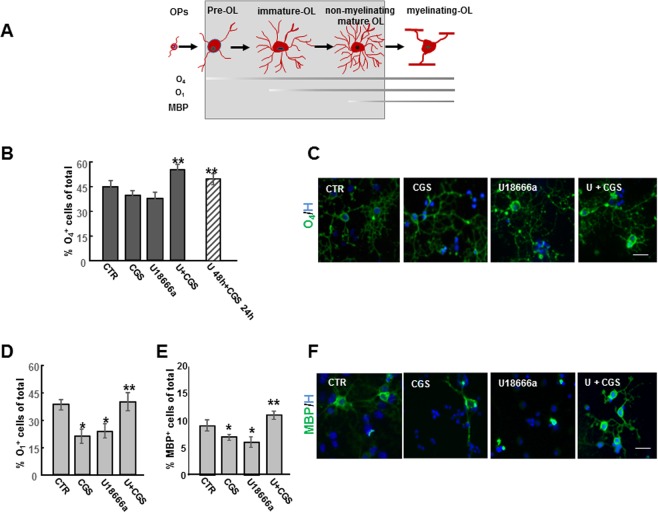
Effects of CGS21680 on maturation arrest induced by U18666a. Schematic representation of morphological and antigenic changes during OL differentiation is shown (**A**). OPs treated for 48 h with 1.25 μM U18666a (U) alone or in combination with 100 nM CGS21680 (CGS) added at the same time or during the last 24 h of incubation, were immunostained for the three developmental markers O_4_, O_1_ and MBP. The percentages of O_4_- and O_1_-and MBP-positive cells are shown in panels (B,D,E). Data are mean ± SEM of 10 microscopic fields per coverslip prepared in duplicate for each condition from 4 to 7 independent experiments (*p < 0.005 vs CTR; **p < 0.05 vs U and for MBP **p < 0.0001 vs U). (**C**–**F**) Examples of OLs immunostained with O_4_ and MBP (green) under different culture conditions. Nuclei were stained using Hoechst 33258 nuclear fluorochrome (blue). (Scale bar = 50 µm).

The consequences of U18666a treatment on OP differentiation were explored by evaluating markers of specific stages of differentiation, such as O_4_ (pre-OL), O_1_ (immature OL) and myelin basic protein (MBP) (non-myelinating mature OL) (Fig. 4A).

A 48 h treatment with U18666a did not affect the percentage of O_4_ positive cells (Fig. 4B) but hampered OP maturation as indicated by the significant decrease in the percentage of O_1_ or MPB positive cells, as compared to control cultures (Fig. 4D,E).

It is known that A_2A_R stimulation affects OP differentiation in a developmental stage-dependent manner^22^. In control conditions (3 DIV) and in agreement with Coppi *et al*.^22^, we observed that CGS21680 decreased the percentage of O_1_ and MBP positive cells with respect to CTR (Fig. 4D,E). However, in U18666a-treated OPs, the presence of CGS21680 protected against U18666a effects on OP maturation, increasing the percentage of cells expressing O_4_, O_1_ and MBP to levels comparable to CTR (Fig. 4B,D,E). Interestingly, CGS21680 was effective even when added during the last 24 h, as shown for O_4_ positive cells (Fig. 4B). The maturation of cells exposed to both U18666a and CGS21680 was confirmed at morphology levels, as shown in Figs 4C and 4F. In particular, MBP staining evidenced a loss of MBP positive arborization in U18666a-treated cells and the regain of complex morphology in cells cultured with U18666a and CGS21680. These data suggested that A_2A_R activation might foster myelination in NPC disease.

**Figure 5:**
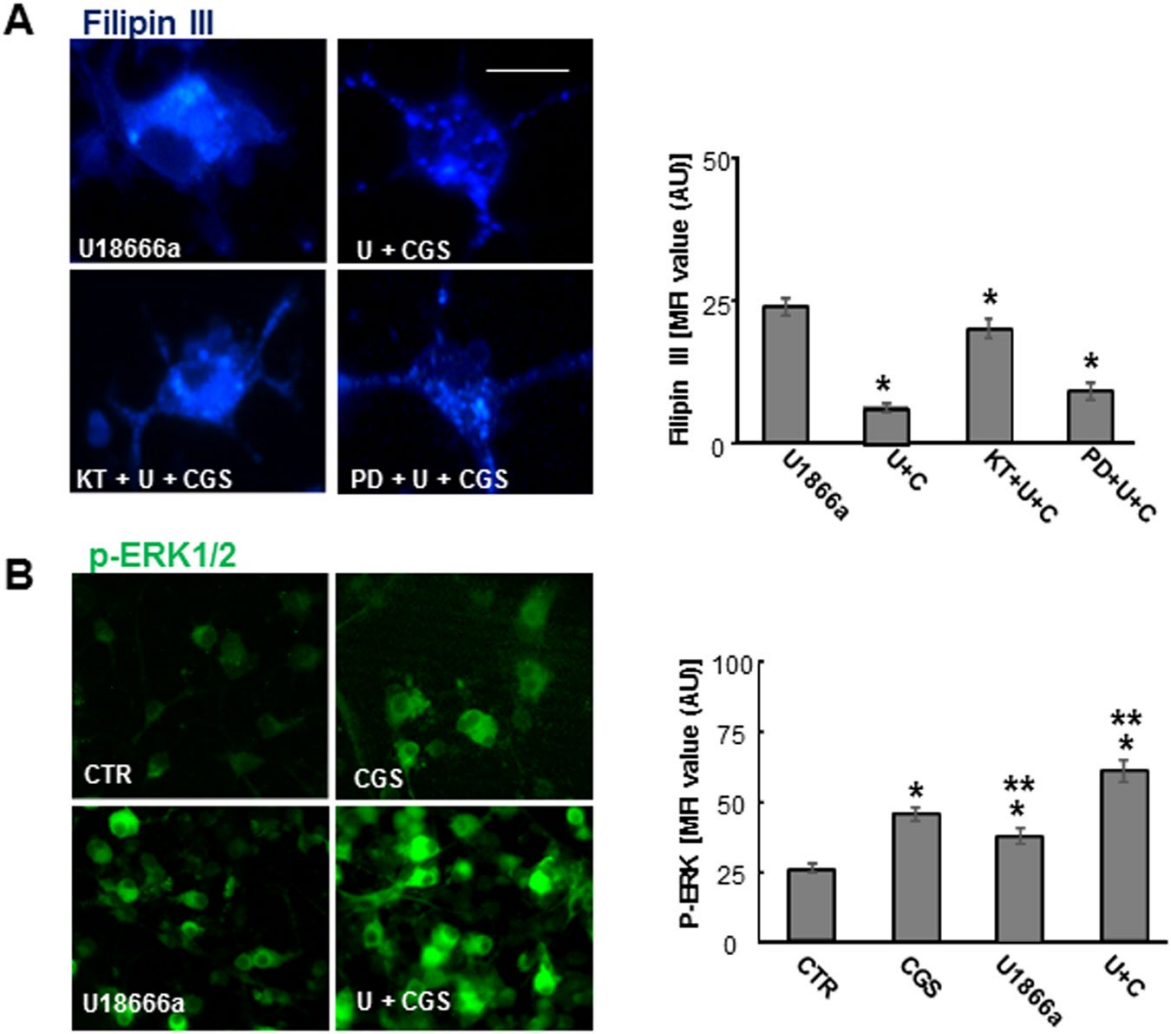
Effects of ERK1/2 inhibitor PD98059 and PKA inhibitor KT5720 on cholesterol accumulation induced by U18666a. (**A**) OPs were treated with U18666a (U) and CGS21680 (CGS) with or without 4 μM PKA inhibitor KT5720 (KT) or 10 μM ERK1/2 inhibitor PD98059 (PD) and cholesterol accumulation evaluated by Filipin III. Mean fluorescence intensities (MFI value) were shown. Data are mean ± SEM of n = 150–200 cells for condition. (*p < 0.001 vs U. Scale bar = 50 µm). (**B**) Examples of OPs treated for 5 min and immunostained with phospho-ERK. Mean fluorescence intensities (MFI value) were shown. Data are mean ± SEM of n = 150–200 cells for condition. (*p < 0.0001 vs CTR; **p < 0.001 vs CGS).

### Activation of cAMP/PKA mediates CGS21680 effects on cholesterol traffic regulation

To gain some insights on the signaling pathways mediating the effects of CGS21680, we studied the involvement of the two main transduction pathways triggered by A_2A_R, namely the activation of extracellular regulated kinase (ERK) 1/2 and of the cAMP/PKA, in contrasting cholesterol accumulation in U18666a-treated OPs. As shown in Fig. 5A, in U18666a-treated cells, the PKA-inhibitor KT5720 (4 μM) completely abolished the reduction of cholesterol accumulation induced by CGS21680 treatment, while the ERK1/2 inhibitor PD98059 (10 μM) did not have any effect. KT5720 and PD98059 were used at concentrations chosen based on preliminary experiments, not affecting cell viability (not shown). Although ERK1/2- activation was not directly involved in cholesterol redistribution, the CGS21680 treatment for 5 minutes induced an increase in protein levels of phosphorylated ERK1/2, the activated form of ERK1/2, indicating that this pathway is functionally active in all experimental conditions (Fig. 5B), but not involved in cholesterol mobilization.

## Discussion

Among the most severe consequences of NPC1-mutation occurring in the CNS is dysmyelination. Both NPC patients and Npc1^−/−^ mice exhibit myelin defects^7,9^, and deletion of NPC1 in both neurons and in OLs impairs OL maturation causing a delayed myelination^6^.

Since no effective treatment is currently available, the identification of new therapeutic targets is an urgent need for NPC1 patients. We previously showed that the stimulation of A_2A_R is able to recover the abnormal phenotype of genetic NPC models such as fibroblasts from NPC1 patients^15^ and neuroblastoma SH-SY5Y and oligodendroglial MO3.13 cell lines transiently transfected with NPC1 siRNA^16^. In this study, we used a pharmacological model of NPC1 OLs to investigate the ability of A_2A_R to rescue the abnormal phenotype and promote OP differentiation into myelin forming cells.

Purified OP cultures from neonatal rat brain are a well-characterized model that recapitulates the process of OL differentiation^23^,^24^. Here we exposed primary cultures of OPs to U18666a, a cholesterol transport inhibitor, to establish a new NPC pharmacological model, and to ascertain the rescuing capability of A_2A_R activation on OL maturation. U18666a has been widely used to induce a NPC-like phenotype in other cell types^17^, but it has never been used in primary OP cultures. Our data demonstrate for the first time that in primary OP cultures, U18666a induces typical features of NPC1 phenotype, including abnormal morphology with multiple vacuole-like structures, mitochondrial depolarization, impaired autophagy and cholesterol accumulation in late endosomal/lysosomal compartments, which is considered the hallmark of NPC1 pathology. These observations indicate that U18666a-treated OPs are good model to screen for potential therapeutic agents for NPC1.

In agreement with a previous study on the expression and the functionality of A_2A_R during development of OLs^22^, we confirmed the greatest expression of receptors at immature stages of development. Furthermore, we observed that in our NPC1 pharmacological model the A_2A_R expression was significantly decreased. Nonetheless, the reduced A_2A_R level in U18666a treated cells was sufficient to trigger a protective effect upon activation by the A_2A_R agonist CGS21680. In a translational perspective, it is of particular interest to observe that either cholesterol accumulation or mitochondrial membrane potential (mMP) alterations were reduced also if cells were exposed to CGS21680 when the damaging effects of U18666a were already in place, (i.e. 24 h after the cholesterol transport inhibitor treatment).

In addition to the well-known disruption of lipid trafficking and cholesterol accumulation in late endosomes and lysosomes, NPC1-deficient cells have increased cholesterol levels in mitochondria, leading to alterations in mitochondrial functions and energy metabolism^3,20,25^. Abnormal mitochondrial morphology, depolarization of mitochondrial membranes, respiratory chain deficit and impaired ATP production have been shown in NPC^−/−^ brain, NPC1-depleted CHO cells and fibroblasts from NPC1 patients^3,5,15^. As a reliable NPC1-cellular model, OP cultures exposed to U18666a exhibited mitochondrial alterations, including mitochondrial fragmentation. In agreement with our previous works in genetic models of NPC1, A_2A_R activation restored mMP and, in addition, reduced mitochondrial fragmentation in U18666a-treated OPs. Using NPC1-deficient and U18666a-treated neuronal cultures derived from human embryonic stem cells, Ordonez and colleagues^13^ showed that excessive activation and impaired progression of the autophagy contribute to abnormal mitochondrial clearance with the accumulation of depolarized and fragmented mitochondria. We found that, in addition to mitochondrial fragmentation, A_2A_R activation rescued the abnormal autophagic flux in U18666a-treated OPs. In details, we observed that A_2A_R agonist CGS21680 decreased cholesterol accumulation in autophagosomes and lysosomes, and reduced abnormal levels of autophagic markers such as Beclin1, LC3 and LAMP2. NPC1 deficient mouse tissue and human fibroblasts treated with U18666a show an increase of Beclin1 and LC3 levels^1,4^, suggesting that altered cholesterol distribution in NPC1 deficient cells promote autophagy as compensation for cholesterol accumulation within late endosomes and lysosomes. However, several reports indicate that in NPC cells the degradation of autosomal cargoes and the fusion of late endosomes to lysosomes and of the autophagosomes to lysosomes are markedly impaired^26–29^. In our NPC1 model, we confirmed an increase of autophagosomes and lysosomes, which was not followed by an increase of autolysosomes, and we demonstrated that the A_2A_R activation prevented these effects.

Importantly, in the present work, we demonstrated that U18666a treatment induces in OPs a maturational arrest, reflecting the abundant OP/pre OL population and the marked decrease of mature OLs observed in prefrontal cortex and in corpus callosum of NPC1^−/−^ mice^9^. In control cultures, in line with studies from Coppi *et al*.^19–22^, CGS21680 induced a decrease in the percentage of O_4_, O_1_ and MBP positive cells with respect to control. In contrast, in 3 DIV cultures, after 48 h of U18666a treatment, CGS21680 overcome the maturation arrest as indicated by the percentage of pre-OL, immature and non-myelinating mature OLs (O_4,_ O_1_ and MBP positive cells, respectively), which returned to control cultures levels. These data were confirmed at morphological levels, as indicated by the restoration of branch complexity, a further index of OPs maturation. As for cholesterol accumulation and mitochondrial depolarization, CGS21680 protected OPs cultures from the differentiation arrest induced by U18666a also when A_2A_R stimulation occurred 24 h after U18666a exposure.

The dual effect of CGS21680 on differentiation, arresting OLs maturation in control cultures and promoting differentiation in U18666a-treated cultures, is in keeping with the opposite effects by CGS21680 recently reported in a model of Huntington’s disease (HD), where it induced opposite effects in striatum of Huntington versus wild-type mice^30^. Although the molecular mechanism of this dual effect in our NPC model has to be clarified, it has to be noted that it meets the physiological needs to maintain a population of quiescent and undifferentiated OPs in normal condition, and promote OPs differentiation in pathologic conditions.

Finally, the analysis of intracellular signaling demonstrated that the PKA pathway is responsible for the A_2A_R-dependent effect on cholesterol accumulation since the PKA inhibitor KT5720, but not the ERK1/2 inhibitor PD98059, prevented the cholesterol redistribution induced by CGS21680 in NPC OPs. In agreement with our previously data^16^, we proposed that the stimulation of A_2A_R, by activating PKA pathway, can revert lysosomal calcium abnormalities and re-establish the correct autophagic flux and cholesterol trafficking. However, differently from NPC1 siRNA-MO3.13 cell line, in U18666a-treated primary OP cultures, the stimulation of A_2A_R was able to trigger both PKA and ERK1/2 transduction pathways, as indicated by the rapid ERK1/2 phosphorylation induced by CGS21680. This difference likely reflects a more advanced state of differentiation of OP cultures. Our findings indicate that A_2A_R can positively affect several typical cell abnormalities of NPC1 OLs and promote the correct process of myelination. The results further support A_2A_R agonists as potential tools to treat the disease. We have recently reported that A_2A_R activation ameliorated motor coordination and cognitive impairment, increased the survival of Purkinje neurons, reduced sphingomyelin accumulation in liver and prolonged the lifespan of the mouse model Balb/c Npc1^nih^ ^31^. The present study suggests that A_2A_R stimulation ameliorates the NPC1 phenotype, by rescuing from neurological abnormalities typical of this disease, including dysmyelination.

## Material and Methods

### Cell cultures

Purified cultures of oligodendrocyte progenitors (OPs) were prepared from newborn Wistar rats (RRID: RGD_13508588; from Charles River) as described^32^ and in accordance with the European Communities Council Directive N. 2010/63/EU and the Italian Law Decree n° 26/2014 (authorization from Ministry of Health: 152/2016-PR). OPs growing on top of mixed glial monolayers were mechanically detached and seeded at the density of 6 × 10^4^ cells/cm^2^ into poly-L-lysine-coated 35-mm diameter plastic culture dishes, 96 well plates, 10/12 mm or 20 mm wide glass coverslips. At 2 h after plating, the culture medium (DMEM with high glucose, BioWest, Fla, USA), supplemented by 10% FCS (Gibco,ThermoFisher Scientific, Inc.) was replaced with a chemically defined serum-free medium, consisting of DMEM/Ham F12 (4:1, BioWest) supplemented with 5.6 mg/ml glucose, 5 µg/ml insulin, 100 µg/ml human transferrin, 100 µg/ml bovine serum albumin, 0.06 ng/ml progesterone, 40 ng/ml sodium selenite, 16 µg/ml putrescine, 50 U/ml penicillin, 50 µg/ml streptomycin and 2 mM glutamine (all products are Sigma, MO, USA), 10 ng/ml human recombinant PDGF-AA and 10 ng/ml human recombinant bFGF (PeproTech EC, Ltd, UK). After 24 or 48 h, cells were exposed to several compounds (U18666a – Calbiochem; CGS21680- Tocris -Bristol, UK; ZM241385- Tocris -Bristol, UK; BAY60-6583- Tocris -Bristol, UK; KT5720- Sigma MO, USA; PD98059- Santa Cruz Biotechnology, Inc, CA) as detailed in figure legends.

### Cell viability

Cell viability was determined by measuring cellular metabolic activity, using the 3-(4,5-dimethylthiazol-2-yl)-2,5-diphenyltetrazolium bromide (MTT, Sigma, Munich, Germany) reduction assay as previously described (Bernardo *et al*., 2009).

### Immunofluorescence

For double staining of primary OPs, the cells were incubated with the oligodendroglia marker O_4_, or O_1_ by using as primary antibodies mouse monoclonal immunoglobulin M (IgM, hybridoma supernatants, custom made, or Millipore, Italia; 1:5 or 1:100, (Cat#MAB344 clone 59 Lot#2343576) respectively), or mouse monoclonal anti-MBP (1:100, Cat#MAB382, RRID:AB_94971, Millipore) and as secondary antibody, fluorescein-conjugated goat anti-mouse IgM or IgG (1:200, Jackson ImmunoResearch Laboratories, Inc, West Grove, PA). The cells were then fixed in 4% paraformaldehyde, permeabilized for 10 min with 0.1% Triton X-100, blocked for 1 h in: 3% BSA, 0.1% Triton X-100 (for LC3 and Beclin 1 labeling) or in 10% goat serum, 0.25% Triton X-100 (for LAMP2 labeling) or in 4% horse serum, 0.1% Triton X-100 (for A_2A_ receptor labeling) and then incubated for 2–3 h at RT with the primary antibodies in the same blocking solution. The primary antibodies used were: anti-A_2A_-receptor (1:100, Cat#05-717, RRID: AB_11213750 Millipore, Temecula, CA), goat polyclonal anti-LAMP2 (1:100, sc8100, C20, lot #E1310, Santa Cruz Biotechnology, Inc, CA), rabbit polyclonal anti-LC3 (ab58610, lot #GR6887-2), anti-Beclin1 (ab55878, lot #888104), (1:100 Abcam, UK) and phospho-extracellular signal-regulated kinase (p-ERK, 1:100, #4370, lot #12 Cell Signaling Technologies). Only for p-ERK staining, cells were incubated with 2.5% horse serum before being incubated for 1 h at 37 °C with the primary antibody; after washing, they were incubated first with biotinylated goat α-rabbit (1:200, Vector Laboratories, Inc, CA, USA) for 45 min at 37 °C and then with Alexa 488 (1:200, Jackson ImmunoResearch Laboratories, Inc, PA) for 1 h at RT. In all other cases, secondary antibodies, Cy3R IgG or FITC IgG polyclonal or monoclonal goat antibodies (1:200, Jackson ImmunoResearch Laboratories, Inc, PA, USA) were used. Validation data for the antibodies are available from the companies. To localize cholesterol within cell membranes, Filipin III (250 μg/ml, Sigma, Munich, Germany), a fluorescent agent known to bind to un-esterified cholesterol, was used. For double staining, cells already processed through primary and secondary antibodies, were treated with a glycine solution (1.5 mg/ml PBS) for 10 min to RT and then exposed to Filipin III for 30 min, in the dark. Nuclei were stained using Hoechst 33258 (5 µg/ml for 20 min, Sigma, Munich, Germany) or propidium iodide (PI, 1:1000 for 5 min RT, Sigma, Munich, Germany). Experiments were at least from three different cultures of OPs, run in duplicate. For cell imaging, at least six fields from each coverslip were captured, and all cells of the field analyzed for fluorescence levels. Coverslips were mounted with Vectashield Mounting Medium (Vector Laboratories, Burlingame, CA), and examined using a Leica DM4000B fluorescence microscope equipped with DFC420C digital camera and Leica Application Suite Software (260RI) for image acquisition. Image acquisition was maintained in the same setting under various experimental conditions. Exposure parameter, saturation, time and gain were set at the beginning of every single group of experiments. For the images obtained from fixed cells, the acquisition informations are: space resolution 2592 × 1944, pixel dimensions 0.340 µm/pixel, image bit depth 8 bitx3 channels. The fluorochromes used are in the range of wavelengths of 495–520 for L5 (green), 552–663 for Y3 (red) and 430–474 (A4) (blue). Every image was acquired with maximum resolution of 400 DPI. The scale bars are indicated in each figure legend. All image analyses were conducted using NIH ImageJ software (http://rsb.info.nih.gov/ij/). Mean threshold fluorescence intensity within a region of interest, delineated by single cell profile, was used to compare expression levels of specific markers. Co-localization of specific markers was analyzed using Pearson’s correlation coefficient^33^. In the case of co-localization ImageJ color lookup table (LUT) pseudo-color was used showing + vePDM with PDM (Product of the Differences from the Mean, i.e. for each pixel: PDM = (red intensity- mean red intensity) × (green intensity − mean green intensity)) value between −0.40 and +0.40. Co-localization channel was built from positive PDM values resulting from both channels pixels that were above the mean.

### Fluorescence video imaging

The potentiometric probe tetramethylrhodamine ethyl ester perchlorate (TMRE) (Cat#87917 Lot#1350313 Sigma-Aldrich) was used to measure mitochondrial membrane potential (mMP) as previously reported^12,15^. Cells were incubated with 30 nM TMRE for 30 min in the dark before recording. The TMRE fluorescence intensity, produced by excitation wavelength 535 nm applied by means of a monochromator (Polychrome II; Till Photonics, Munich, Germany) and observed at emission wavelength 590 nm, was collected through an oil immersion objective (Olympus: 40X, 1.35 NA) and recorded by a CCD cooled digital camera (PCO; Sensicam, Kelheim, Germany) mounted on an inverted microscope (Axiovert 135, Zeiss; Germany). Acquisition information are: space resolution 1280 × 1024, pixel dimensions 0.184 µm/pixel, image bit depth 12 bit; experimental conditions: room temperature; medium composition (140 NaCl, 5 KCl, 2.5 CaCl2, 1 MgCl2, 10 glucose, 10 Hepes, pH 7.4). Images were recorded and analyzed by Imaging Workbench 6.0 software package (Indec BioSystems, Santa Clara, CA).

The Mito Tracker green probe (Cat#M7514 Lot#431620 Thermo Fisher Scientific Inc.) was used to measure mitochondrial length. Cells were incubated with 40 nM MitoTracker green for 30 min. The fluorescence intensity, produced by excitation wavelength 488 nm obtained by an Argon Ion Laser and observed at emission wavelength 495–550 nm, was collected through a planapo objective (Olympus) 60 × oil A.N.1.42 and recorded by a FV1000 confocal microscope (Olympus, Tokyo, Japan) equipped with a confocal spectral imaging system (OlympusFluoview 1000). Images recorded have an optical thickness of 0.5 mm. Images were recorded at 640 × 640 pixels, 16 bit, corresponding to a dimension of 140.8 × 140.8 mm for each frame and each pixel has dimension of 0.22 × 0.22 mm. Experimental conditions: room temperature and for medium composition see above. Images were analyzed by NIH ImageJ software (http://rsb.info.nih.gov/ij/). The mitochondria were divided into categories based on their length as previously described^34^. The TMRE fluorescence intensity was normalized as the percentage of each treatment group over the control. Images were analyzed offline by measuring the emission value in regions of interest or along line profiles. Data were collected from a minimum of 3 experiments for each condition and shown in bar graphs as mean ± SEM, with n = number of observations.

### Western blot analysis

Cells were homogenized on ice in RIPA buffer (phosphate-buffered saline, 1% NP-40, 0.5% sodium deoxycholate, 0.1% sodium dodecyl sulpate, protease inhibitors) and centrifuged at 12000 g for 20 min, 4 °C. 50 µg (for Beclin1 and LC3A/B) of protein were separated by 8% SDS-PAGE. Proteins were transferred to polyvinylidene difluoride membranes by electroblotting for 1 h at 4 °C, and membranes incubated for 1 h in T-TBS (50 mM Tris–HCl, 150 mM NaCl, 0.05% Tween- 20, pH 7.4), with 5% non-fat milk. Blots were incubated overnight at 4 °C with the following antibodies: rabbit anti-Beclin1 or anti-LC3A/B (1:400 Abcam, Cambridge, UK) and mouse anti-β-actin (1:20000, Cat#A2228, RRID: AB_476697 Sigma-Aldrich), used as internal control. After incubation with HRP-conjugated antibodies (Jackson Immunoresearch), immunoreactive bands were revealed by enhanced chemiluminescent substrate onto X-ray films. The bands, were analyzed using NIH ImageJ software (http://rsb.info.nih.gov/ij/).

### Statistical analysis

Data were expressed, as means ± SEM. Statistical significance was evaluated using two-sided Student’s t test to compare two experimental groups. Numbers of independent experiments are indicated in the figure legends; p < 0.05 was accepted as statistically significant. The experimental procedures and statistical analysis of the data presented in this study followed the methodologies and standards generally used in *in vitro* studies. In all experiments, the sources of variability as well as any residuals deriving from random errors of the replicates were well controlled, as demonstrated by the low SEM. Bonferroni correction was adopted to account for multiple comparisons. Five comparisons were considered (CGS21680 vs CTR, U18666a vs CTR, CGS21680 + U18666a vs CTR, CGS21680 + U18666a vs CGS21680 and CGS21680 + U18666a vs U18666a). Normality of distribution was assessed by Shapiro Wilk test.

## Supplementary information

Supplementary data
