# Supplementary material for: Adenosine A2A receptor stimulation restores cell functions and differentiation in Niemann-Pick type C-like oligodendrocytes
Source: Sci Rep. 2019 Jul 5;9:9782. doi: 10.1038/s41598-019-46268-8 (PMC6611770; doi:10.1038/s41598-019-46268-8)
Supplement: Supplementary file 1 — Supplementary data [file 41598_2019_46268_MOESM1_ESM.docx]

**Supplemental Data**

**Adenosine A_2A_ receptor stimulation restores cell functions and differentiation in Niemann-Pick type C-like oligodendrocytes**

Chiara De Nuccio, Antonietta Bernardo, Antonella Ferrante, Rita Pepponi, Alberto Martire, Mario Falchi, Sergio Visentin, Patrizia Popoli and Luisa Minghetti

**Figure 1S**


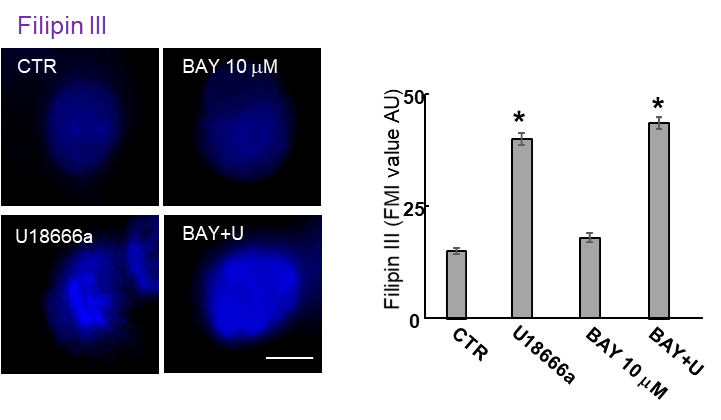


**The A_2B_R agonist BAY60-6583 does not rescue the effects of U18666a on cholesterol accumulation.**

The OPs were treated for 48h with U18666a (U) alone or with 10 μM BAY60-6583 (BAY). Cholesterol was labeled with Filipin III. Mean fluorescence intensities (MFI value) of Filipin III are shown. Data are mean + SEM, n=180-250 cells for condition (*p<0.0001 vs CTR). Scale bar=50 µm

**Adenosine A_2A_ receptor stimulation restores cell functions and differentiation in Niemann-Pick type C-like oligodendrocytes**

Chiara De Nuccio, Antonietta Bernardo, Antonella Ferrante, Rita Pepponi, Alberto Martire, Mario Falchi, Sergio Visentin, Patrizia Popoli and Luisa Minghetti

**Figure 2S**


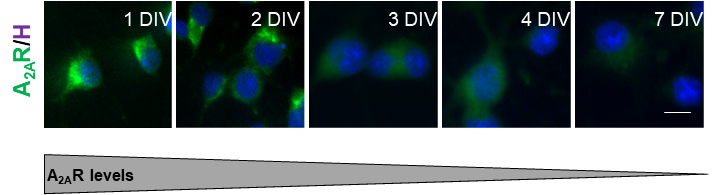


**The expression of A_2A_R is modulated during OP differentiation.**

The A_2A_R expression was evaluated by immunofluorescence. Time course of A_2A_R expression in OPs under control conditions is shown. Scale bar=50 µm.

**Adenosine A_2A_ receptor stimulation restores cell functions and differentiation in Niemann-Pick type C-like oligodendrocytes**

Chiara De Nuccio, Antonietta Bernardo, Antonella Ferrante, Rita Pepponi, Alberto Martire, Mario Falchi, Sergio Visentin, Patrizia Popoli and Luisa Minghetti

**Figure 3S**


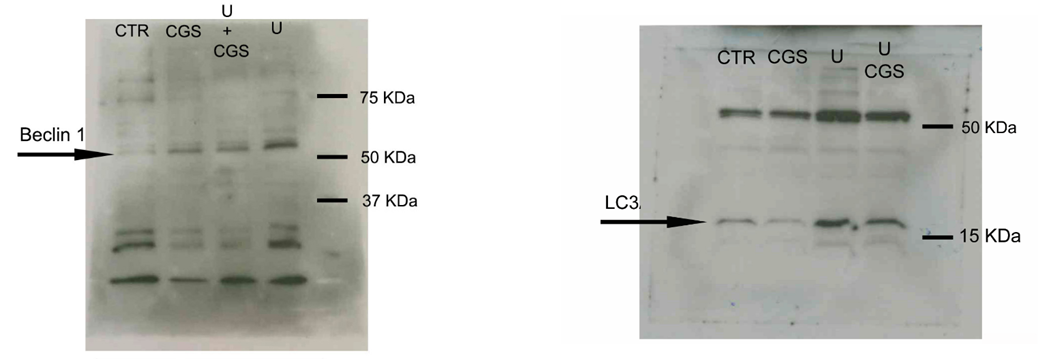


Representative Western blot (WB) of three different experiments for Beclin1 and LC3 of OP cultures treated for 48h with U18666a (U) and CGS21680 (CGS) were shown. The images represent full-length blots.

WB experiments were performed to confirm immunofluorescence (IF) data. The antibodies used for WB experiments are the same as those used for IF experiments. In IF experiments the antibodies were characterized for their specificity. The anti-LC3 Ab (ab58610, lot #GR6887-2) and the anti-Beclin1 Ab (ab55878, lot #888104) were selected to carry out our experiments because of their specific characteristics as reported in the datasheet.
